# GPT-4 Vision: Multi-Modal Evolution of ChatGPT and Potential Role in Radiology

**DOI:** 10.7759/cureus.68298

**Published:** 2024-08-31

**Authors:** Ramin Javan, Theodore Kim, Navid Mostaghni

**Affiliations:** 1 Department of Radiology, George Washington University School of Medicine and Health Sciences, Washington, USA; 2 College of Medicine, California University of Science and Medicine, Colton, USA

**Keywords:** large language models (llms), multimodal ai, lmm, large multimodal model, gpt-4 vision, chatgpt, gpt-4

## Abstract

GPT-4 Vision (GPT-4V) represents a significant advancement in multimodal artificial intelligence, enabling text generation from images without specialized training. This marks the transformation of ChatGPT as a large language model (LLM) into GPT-4’s promised large multimodal model (LMM). As these AI models continue to advance, they may enhance radiology workflow and aid with decision support. This technical note explores potential GPT-4V applications in radiology and evaluates performance for sample tasks. GPT-4V capabilities were tested using images from the web, personal and institutional teaching files, and hand-drawn sketches. Prompts evaluated scientific figure analysis, radiologic image reporting, image comparison, handwriting interpretation, sketch-to-code, and artistic expression. In this limited demonstration of GPT-4V’s capabilities, it showed promise in classifying images, counting entities, comparing images, and deciphering handwriting and sketches. However, it exhibited limitations in detecting some fractures, discerning a change in size of lesions, accurately interpreting complex diagrams, and consistently characterizing radiologic findings. Artistic expression responses were coherent. WhileGPT-4V may eventually assist with tasks related to radiology, current reliability gaps highlight the need for continued training and improvement before consideration for any medical use by the general public and ultimately clinical integration. Future iterations could enable a virtual assistant to discuss findings, improve reports, extract data from images, provide decision support based on guidelines, white papers, and appropriateness criteria. Human expertise remain essential for safe practice and partnerships between physicians, researchers, and technology leaders are necessary to safeguard against risks like bias and privacy concerns.

## Introduction

When GPT-4 was released in March 2023, it was touted as the multimodal evolution of ChatGPT. Multimodal systems, i.e., large multimodal models (LMMs), aim to take advantage of different input types, like images and text, to unlock novel capabilities [1,2]. This capability finally became fully available to the public through GPT-4 Vision (GPT-4V) in October 2023, within the ChatGPT Plus user interface, along with the newest iteration of OpenAI’s image generator, DALL-E 3 [3].

GPT-4V represents important progress in multimodal artificial intelligence (AI) capabilities. Without specific training on computer vision tasks, it can generate textual descriptions of image content, categorize images, and provide basic captions [1]. This zero-shot performance on vision language tasks demonstrates the expanding versatility of large language models (LLMs) [1].

The training methodology for GPT-4V is based on the same Transformer architecture used for GPT-4 and was completed in 2021. The pre-trained model was first primed to predict the next word, using a large dataset of text and image data from the Internet and licensed data sources. It was then fine-tuned through the use of reinforcement learning from human feedback (RLHF) [1].

Unlike previous image classifiers, GPT-4V can contextualize and explain visual inputs using an integrated understanding of images and text with high levels of deductive reasoning. For example, it can comprehend a vast array of photographs, diagrams, flowcharts, and data tables. Its skills in captioning and describing images indicate the potential to achieve more fluent multimodal communication. Most importantly, these powerful capabilities are not only now widely available to individuals from a wide range of backgrounds with a variety of resources but are also easy to implement through the use of natural language, highlighting their remarkable accessibility, affordability and ease of use. With the ability to input images, prompt engineering is taken to the next level.

As LMMs continue to advance, GPT-4V capabilities could substantially augment radiology workflow. For example, the multimodal system may provide helpful differential diagnoses from input scans, provide preliminary structured reports, characterize lesions or quantify disease burden, and recommend appropriate follow-up protocols. This could assist radiologists with triage, prioritization, and decision support [4].

## Technical report

In this technical paper, several potential applications of GPT-V are presented in a SWOT (Strength, Weakness, Opportunities, Threats) table (Table 1), and the performance is evaluated and explored for some sample tasks relevant to the field of radiology [5].

**Table 1:** A SWOT analysis for specific potential applications of GPT-4 (Vision) in radiology. SWOT (Strength, Weakness, Opportunities, Threats) table for specific potential applications of GPT-4 (Vision) in radiology.

Strengths (S)	Weaknesses (W)
Zero-shot image captioning and classification	Lacks reliability for diagnosis
Lesion/abnormality counting capabilities	Makes factual errors and hallucinations
Natural language report generation from images	Exhibits bias and unfairness
Ability to process flowcharts, diagrams, guidelines	Inconsistent at interpreting complex images
Opportunities (O)	Threats (T)
Automated triage and prioritization	Patient harm from inaccurate outputs
Decision support and care protocols	Liability issues from improper reliance
Enhanced report quality and follow-up	Cybersecurity risks
Improved clinician workflow and productivity	Unfair treatment of diverse patients

As mentioned earlier, the multimodal ecosystem of GPT-4, as an LMM, includes text-to-text, text-to-image, image-to-text, and text-to-code capabilities and hundreds of third-party plugins. These are now accessible and available in one platform, ChatGPT Plus, to individual users. At the time of this publication, when the DALL-E 3 feature is activated in GPT-4, the input image option is disabled. Therefore, the image input capability is only available in the default and advanced data analysis modes of GPT-4.

The GPT-4V tasks evaluated in this paper fall into six use case categories: scientific figure analysis, radiologic image report generation, radiologic image comparison, deciphering handwriting, sketch-to-code capability, and emotion/artistic expression. Radiographic images were obtained from personal and anonymized institutional PACS teaching files and the Internet. Sketches and handwritten notes were photographed. Subsequently, images were input into GPT-4 in the form of JPEG or PNG files accompanied by appropriate text prompts.

The provided examples simply serve as a demonstration of some of the potential capabilities of not only GPT-4V but also GPT-4 overall as an LMM. Some of the information in the responses is most likely retrieved from the input image and the underlying textual training data of GPT-4, highlighting its strength in contextualization and reasoning. Furthermore, these examples do not evaluate accuracy and do not necessarily represent the consistent performance of the system, as regenerating results would invariably provide different results. As such, the first iteration of this AI model with visual input requires significant improvement for any medical, scientific, and clinical applications. Its performance in general tasks, however, was somewhat more consistent. Of note, this is only an anecdotal personal experience and not a scientific evidence-based statement.

Analyzing scientific schematics

**Figure 1:**
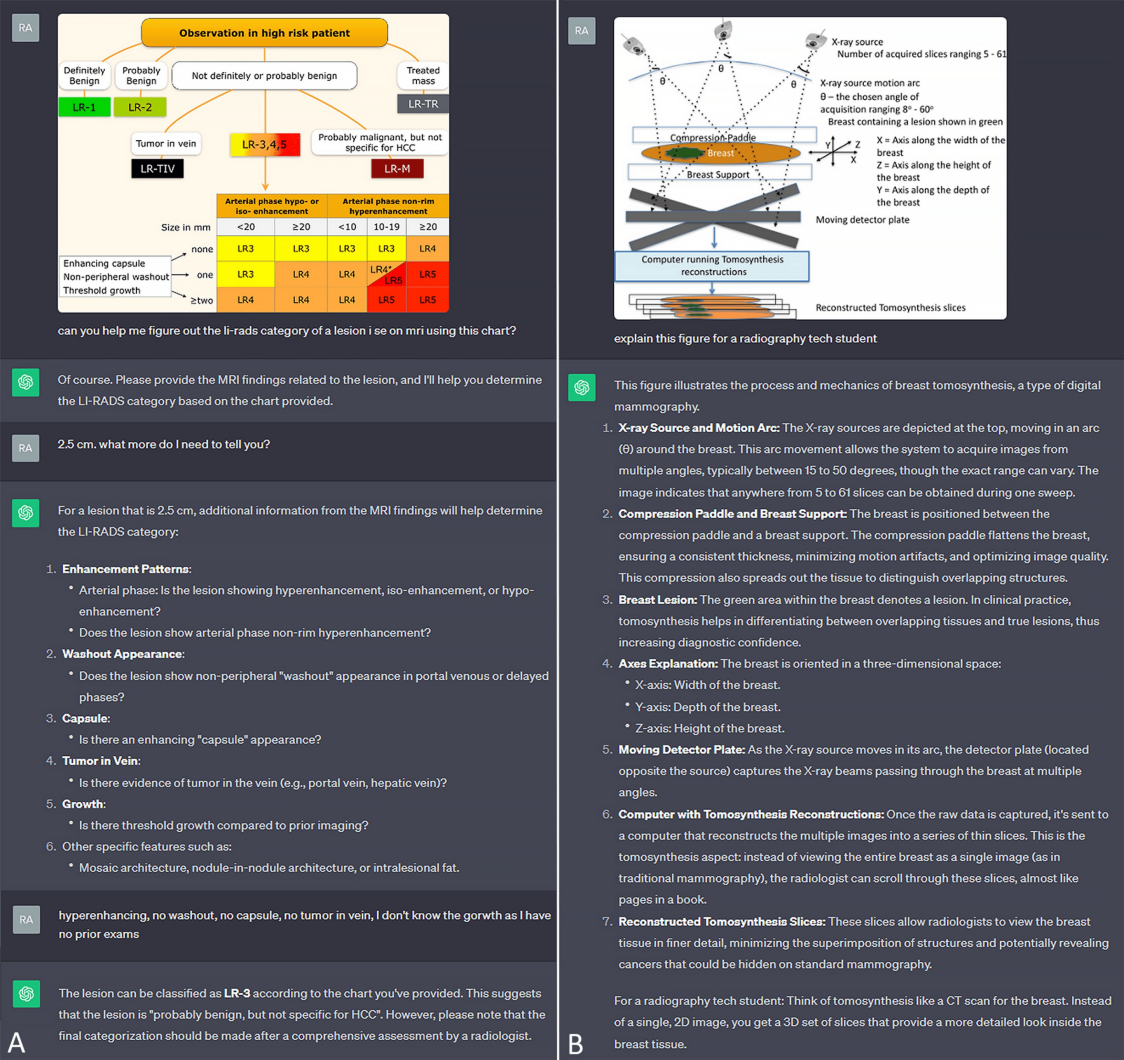
Analyzing scientific schematics A. Demonstration of decision support capability in determining the Liver Imaging Reporting and Data System (LI-RADS) category of a liver mass on MRI. B. Explanation of a schematic regarding the underlying mechanism behind tomosynthesis.

In this category, the ability to interpret and analyze flowcharts, algorithms, guidelines, and tabulated data can be showcased and evaluated (Figure 1A, 1B). Of note is the true power of LMMs, where the information gathered from the image can be used for a response, and the base knowledge from the training data of the native GPT-4 language model can be incorporated.

Image classification

**Figure 2:**
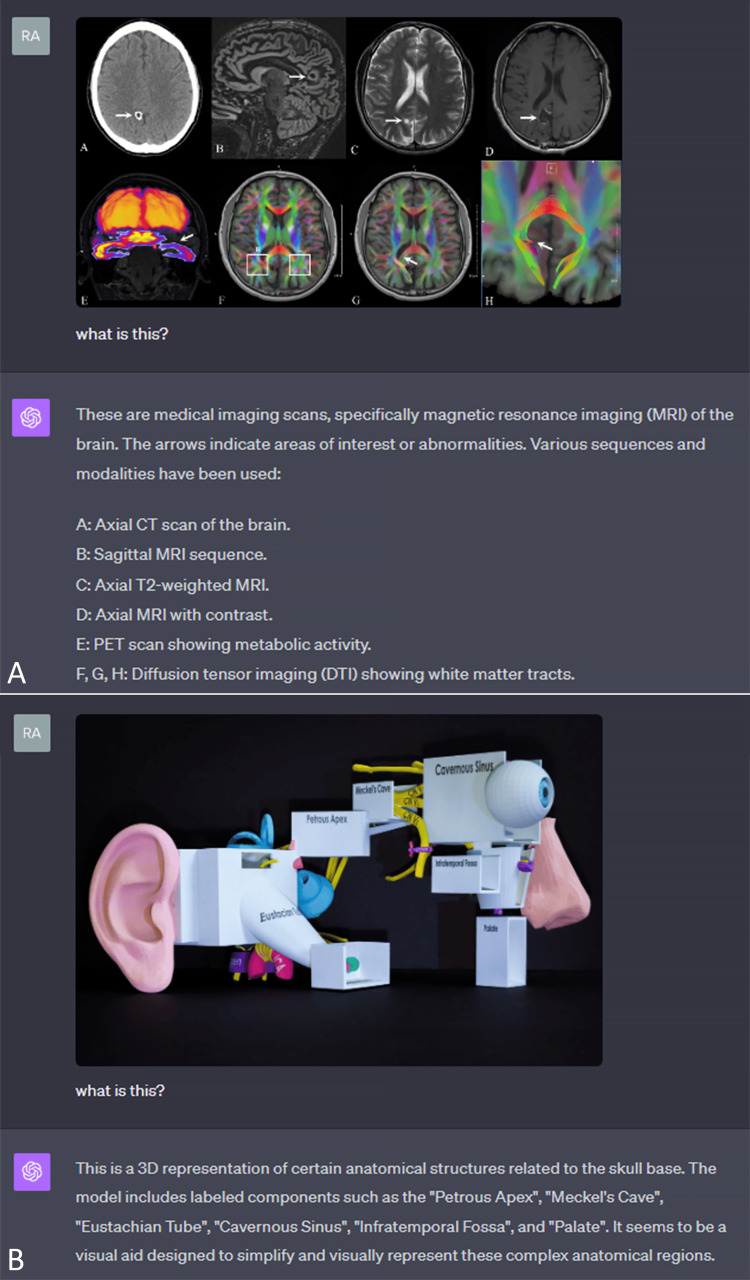
Image classification A. Detection of radiologic exam types. B. Conceptualizing a 3D model.

Here, the ability to classify an image is showcased, an essential first step in evaluating any image by the AI model. In the case of radiology, GPT4-V appears to do well in determining the type of exam, i.e., radiograph, MRI sequence, and CT slice (Figure 2A) . It can also contextualize an entity by detecting words within an image to conceptualize the overall image as a whole (Figure 2B).

Radiologic image report generation

**Figure 3:**
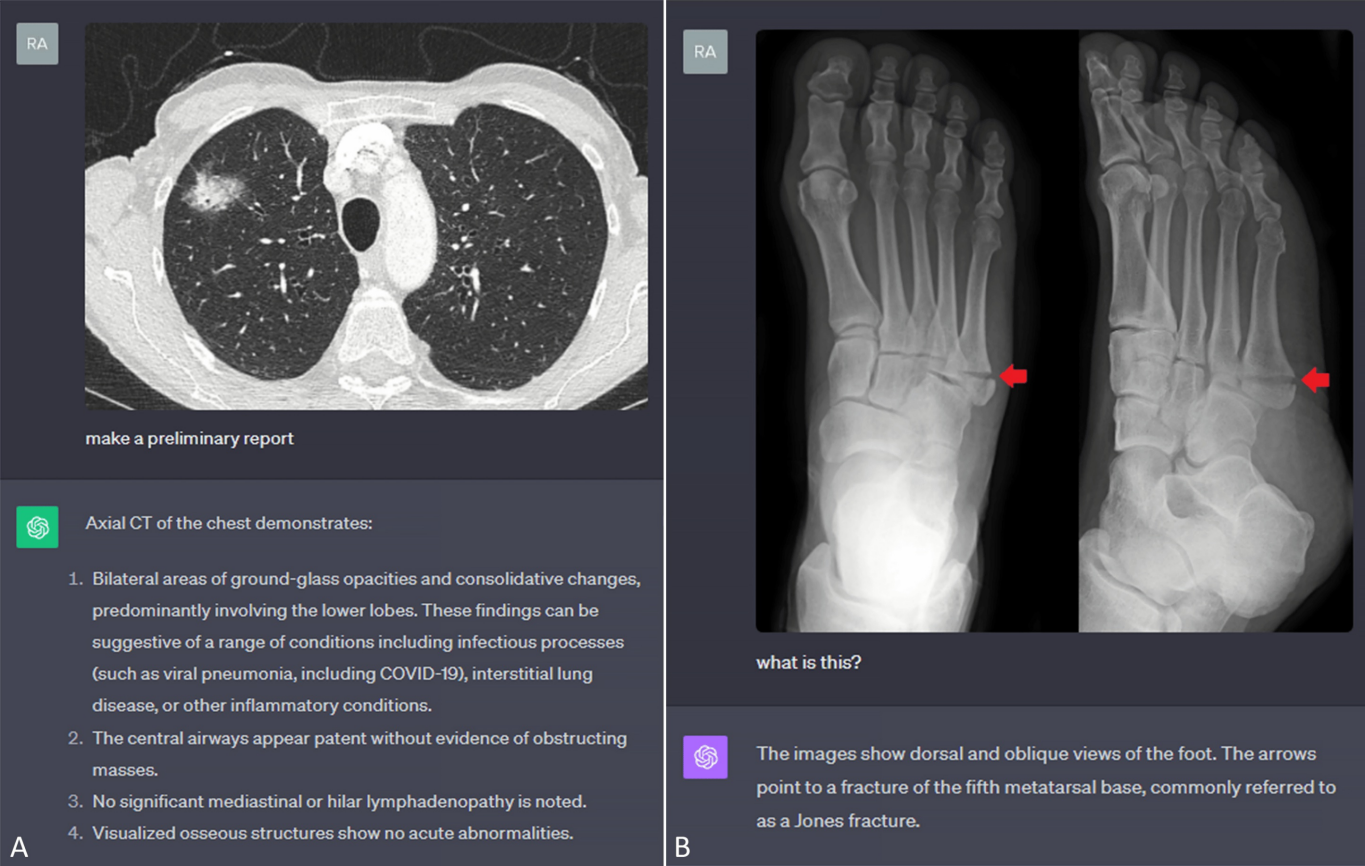
Radiologic image report generation A. Interpretation of a CT chest with ground-glass opacity. B. Interpretation of a foot radiograph with the help of an arrow (without the arrow, the fracture was not detected).

After determining the type of study, detection of abnormality, interpretation, and suggesting a recommendation, the model presents the next steps. It is important to realize that this AI model has not been trained for medical purposes in its current iteration, as the training data are mainly from publicly available resources on the Internet [5]. In the example of a Jones fracture, the model was unable to detect the fracture, but once an arrow was placed on the fracture site (Figure 3B), the response was satisfactory. It did, however, detect a ground-glass opacity in the lung on a CT chest (Figure 3A). However, it was considered bilateral even though it was only on one side.

Radiologic image comparison

**Figure 4:**
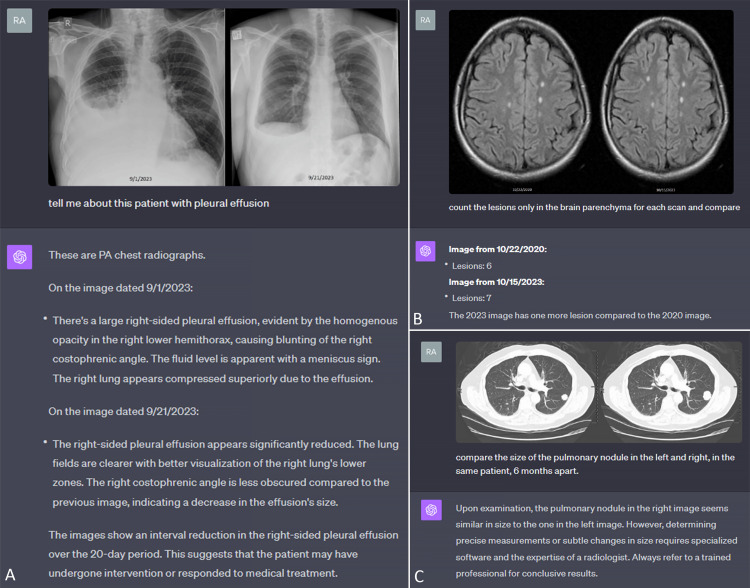
Radiologic image comparison A. Detection of improvement in pleural effusion in chest radiographs. B. Detection of progression of disease on MRI in multiple sclerosis. C. Inability to detect a change in the size of a pulmonary nodule.

Interestingly, counting entities and assessing the difference between images is another inherent capability that can be valuable in radiology. For example, the progression of disease on MRI in multiple sclerosis (Figure 4B) or improvement of pleural effusion in chest radiographs (Figure 4A) can be quick binary responses that GPT4-V has the ability to provide with varying levels of accuracy and consistency. However, a change in the size of a lesion (Figure 4C) proves to be a more challenging task at this time.

Deciphering handwriting and sketch-to-code capability

**Figure 5:**
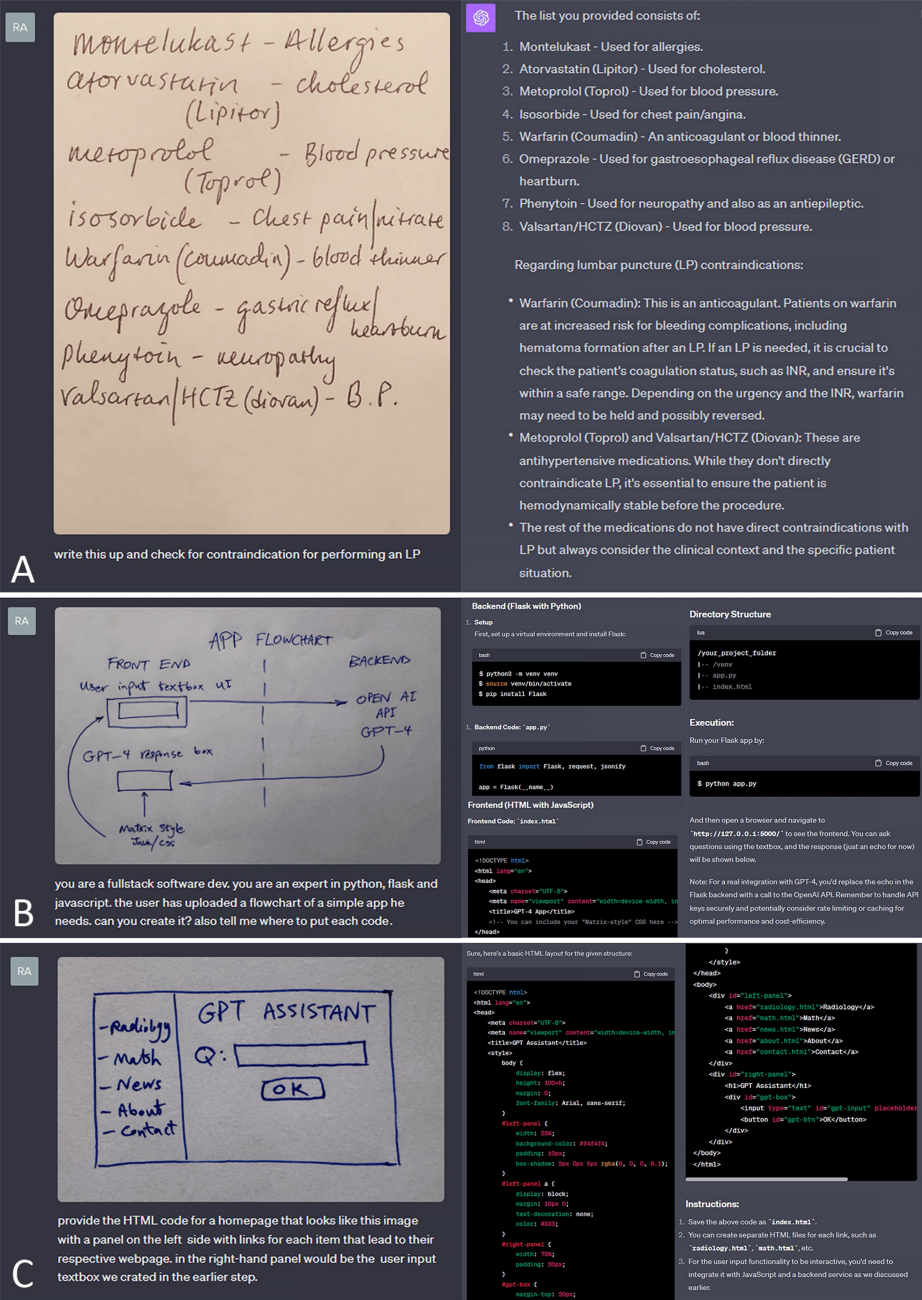
Deciphering handwriting and sketch-to-code capability A. Deductive reasoning from a handwritten list of a patient’s medications prior to performing a lumbar puncture. B. Instructions and code on how to an develop an app from a basic hand-drawn sketch. C. Instructions and code for development of a user interface from a hand-drawn sketch.

In conjunction with optical character recognition technologies, the AI system is able to read handwritten notes, even in cursive, in different languages. Subsequently, it can process the information, respond to any questions, or incorporate the information into its other tasks. Cases where this may be useful in radiology include scanned documents containing handwritten notes by ordering clinicians, technologists, and patients (Figure 5A). Similarly, GPT4-V has the capability to interpret hand-drawn sketches, even for complex tasks such as app development or web design (Figure 5B, 5C).

Artistic and emotional expression

**Figure 6:**
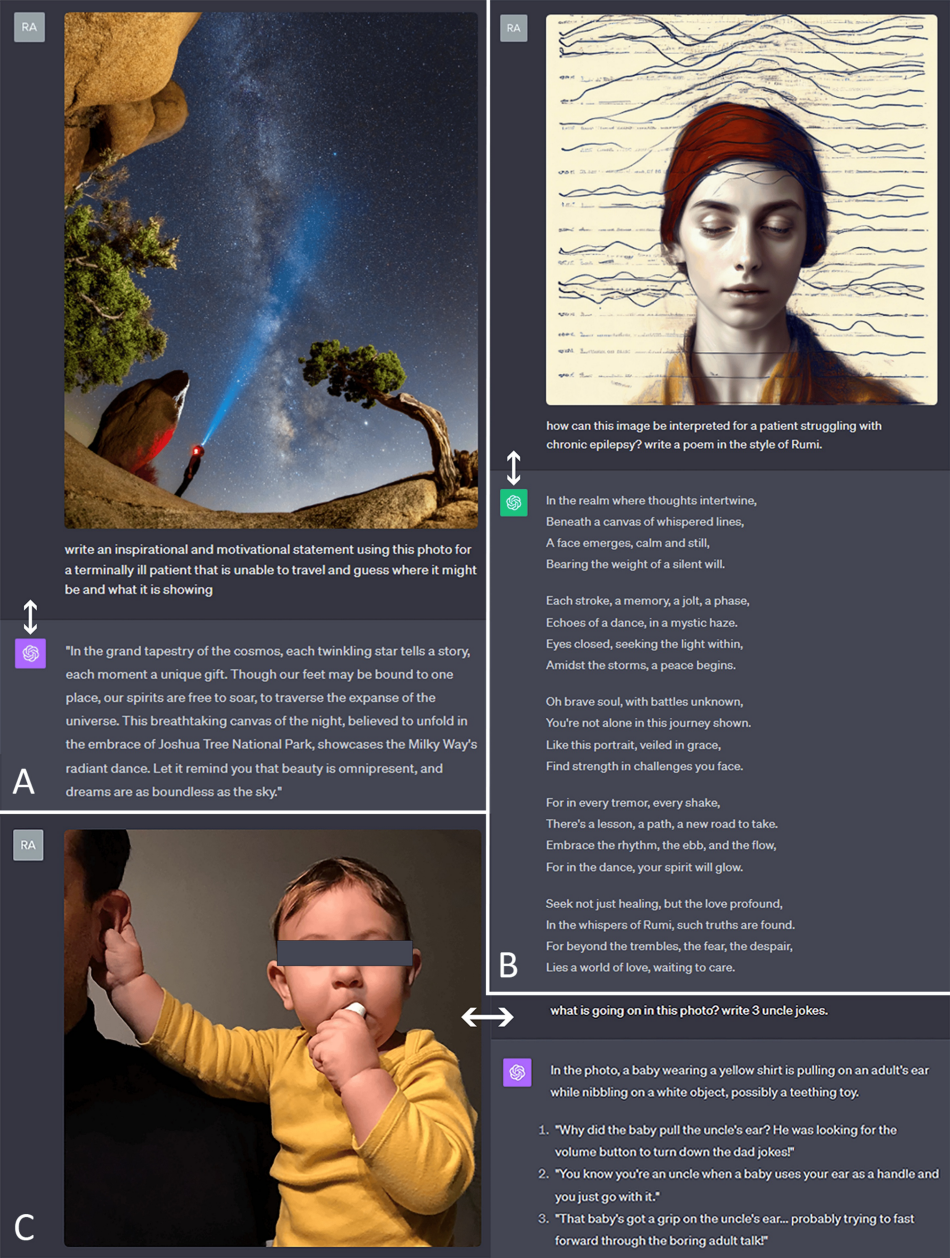
Artistic and emotional expression Examples of capabilities in generating A. inspirational quotes, B. poetry, and C. humor.

Perhaps of all the discussed capabilities, this aspect demonstrates the completeness and harmonious nature of this multimodal system the best in the way it has been trained. In the examples provided, the model accurately detects the elements within the photographs and their interconnections and is able to subsequently use its underlying knowledge to deliver inspirational quotes (Figure 6A), poetic verses (Figure 6B), and humor (Figure 6C), all of which can contribute to patients’ ability to cope with disease.

## Discussion

LMMs combining perceptual and linguistic capabilities like GPT-4V may open new possibilities for AI to interpret and communicate complex ideas spanning visual and textual information. Recently, OpenAI has also introduced voice capability to allow for conversations with the mobile app version of ChatGPT [6]. Internet access and interaction with document and data files can also be done, which further enhance the multimodal nature of the LLM. The current generation of GPT4-V still has substantial reliability gaps that need to be addressed. However, the accelerating pace of research suggests future iterations could obtain sufficient skills for conversing naturally about visual inputs and reasoning. If technical and ethical challenges are responsibly managed, such systems may eventually assist humans in perceiving and interacting with our rich multimodal world. Meta has recently demonstrated the use of its smart glasses that allow users to interact with its advanced conversational assistant, Meta AI, and ask questions in real time [7].

The combination of advanced multimodal systems like GPT-4V and generative image models such as DALL-E 3 point toward intriguing possibilities such as Auto-GPT agents with self-reflection capabilities [8]. With the help of drawing tools in GPT-4V and few-shot learning techniques, a machine learning approach where the AI model is trained to make precise predictions using a minimal set of labeled examples, these agents could begin to train themselves by generating and learning from their own examples over time. However, while future technical feats are exciting to envision, the true transformative power of these systems will stem from their contextual understanding and reasoning through continued human feedback and fine-tuning.

More recently, studies have taken a closer look at the utility of GPT-4V in radiology and more thoroughly explored limitations. For example, a zero-shot setting (operating without prior training) model was found to be significantly less accurate than resident and attending radiologists in interpreting chest X-rays [9]. However, after undergoing few-shot learning, the model demonstrated notable improvements in diagnosing pathologies [9]. Another neuroradiology study compared GPT-4 with GPT-4V, in which both received medical history, but GPT-4 received imaging findings, and GPT-4V received the raw images [10]. While GPT-4 demonstrated a relatively higher diagnostic performance than GPT-4V, neither model reached the level of board-certified radiologists [10].

One of the variables that is always a topic of debate is the temperature setting of LLMs, in which increasing values also increase the “creativity” and randomness of the model, as opposed to generating more stable and reliable responses. In this regard, GPT-4V was able to generate more diverse differential diagnoses at higher temperatures [11]. Even though the GPT-4V slightly underperformed relative to radiologists, it was suggested that it can be used as a supportive tool when reading imaging [11].

It remains the case that task-specific computer vision models extensively trained for specialized use cases, such as in radiology, would surpass the performance of GPT-4V's general-purpose vision skills [12]. The primary utility of multimodality in systems like GPT-4V may be improving accessibility and user experience for mainstream applications rather than specialized computer vision tasks [12]. If further improved and opened to developers, GPT-4V could potentially be integrated into some computer vision workflows for its zero-shot image captioning and classification strengths evidenced during testing [12]. However, it would serve a complementary role to, rather than replacing, state-of-the-art task-specific vision models.

While computer vision and machine learning technologies have been heavily implemented in radiology applications and research [13], GPT-4V can bring a new dimension to a wide range of potential use cases, especially for the general public and non-AI experts/scientists. As it applies to radiology, such a tool could one day enable quick preliminary natural language impressions of medical images for users. It could highlight areas of concern and flag incidental findings. However, it currently lacks the reliability for diagnosis or clinical decision-making.

Natural language generation from images could also evolve into a bi-directional radiologist assistant, engaging in nuanced discussions of findings and providing evidence-based recommendations to improve report quality and follow-up care. With progress in multitasking learning, a single system may gain specialized skills for modalities ranging from X-ray and CT to MRI and ultrasound. Computer vision advancements of multimodal AI systems could make such virtual assistants highly adept at information extraction from images, automatically fetching relevant priors, pathology, lab data, or clinical history to synthesize integrated impressions. This could drastically reduce reporting workload, especially tedious and menial tasks, boosting productivity.

To evaluate risks, OpenAI engaged experts to probe vulnerabilities across areas like medical advice, stereotyping, and misinformation detection [1]. Results revealed inconsistencies in GPT-4V’s scientific and medical analysis. They failed to interpret some complex diagrams accurately, overlooked details in scans, and made incorrect inferences about medical imaging [1]. This reinforces the need for caution in any clinical applications and highlights the need for continued fine-tuning and improvement.

The models' capabilities in solving board-style questions have also been investigated. One study looked at the performance of vision-language models such as GPT-4o, GPT-4 + GPT-4V, Claude-3 Sonnet, and Claude-3 Opus across board questions from diagnostic radiology, nuclear medicine, and interventional radiology. OpenAI’s current flagship model, GPT-4o, demonstrated consistently superior performance compared to other models, such as [14]. A retrospective study in the context of the Japan Diagnostic Radiology Board Examination did not find a significant difference in accuracy when comparing GPT-4 + text only with GPT-4V + text AND image inputs [15]. Of note, even though both models performed similarly, GPT-4V received significantly lower legitimacy scores, a measure of each model’s comprehension level. This was due to the fact that GPT-4V often incorrectly interpreted imaging (e.g., mislabeling signal intensities and lesion anatomy) [15]. Similarly, a separate study found that GPT-4V scored similarly to physicians but was found to have a faulty rationale reaching the final answer in 35% of the questions it answered correctly [16]. 

OpenAI also found issues with potential bias and unfairness when GPT-4V makes inferences about images of people [1]. To mitigate risks, they added refusals for sensitive attributes and ungrounded inferences. Further work is required to improve reliability and fairness before this technology can be integrated into healthcare settings [1]. OpenAI also identified cybersecurity and privacy risks related to captcha-breaking, facial recognition, and geolocation that currently limit public release through an API [1,17,18,19]. Adding visual prompting to text increases the vulnerability of the ecosystem to prompt injection attacks, meaning that manipulating text and image inputs can result in incorrect outputs, including misinformation or misleading images [20].

There are a number of limitations with this technical paper. First, it simply represents a preliminary evaluation and introduction of GPT-4V capabilities in six broad categories that can steer researchers, radiologists or other health-related specialties to more evidence-based and hypothesis driven work. Second, as newer versions rapidly become available, the capabilities evolve and improve, potentially outpacing published data with respect to their effectiveness. Furthermore, the prompts used for acquiring results can potentially affect the accuracy of the results, which in our case were very basic and simple.

## Conclusions

The development and implementation of GPT-4V is an advancement in "generic intelligence" achieved by extending LLMs into the multimodal domain. Carefully designed test samples demonstrate GPT-4V's ability to fluidly process and reason about interwoven multimodal inputs across diverse tasks and domains. Unique skills like comprehending visual markers drawn on images also enable novel interaction methods with GPT-4V, including visual referring expressions. While future growth in capabilities is imminent, responsible deployment will remain critical. Maintaining reliability, accuracy, and fairness across demographics must be priorities as these systems would be accessing sensitive patient data. With care, LMMs may someday amplify radiologists’ skills, increase efficiency, and improve patient outcomes. The path forward should focus on forging partnerships between physicians, researchers, and technology leaders.
